# Advanced Imaging for Localized Prostate Cancer

**DOI:** 10.3390/cancers16203490

**Published:** 2024-10-15

**Authors:** Patrick Albers, Adam Kinnaird

**Affiliations:** 1 Division of Urology, Department of Surgery, University of Alberta, Edmonton, AB T6G 1Z2, Canada; palbers@ualberta.ca; 2 Alberta Prostate Cancer Research Initiative (APCaRI), Edmonton, AB T6G 1Z2, Canada; 3 Cancer Research Institute of Northern Alberta (CRINA), Edmonton, AB T6G 2E1, Canada; 4 Alberta Center for Urologic Research and Excellence (ACURE), Edmonton, AB T6G 1Z2, Canada; 5 Department of Oncology, University of Alberta, Edmonton, AB T6G 1Z2, Canada

**Keywords:** prostate cancer, localized, diagnosis, prostate biopsy, PSMA PET, micro-ultrasound, MRI, imaging, targeted prostate biopsy

## Abstract

**Simple Summary:**

This review paper explores advanced imaging technologies for prostate cancer because traditional methods often miss or misclassify cancers. The aim of this study is to provide a comprehensive overview of three key technologies, MRI, micro-ultrasound, and prostate-specific membrane antigen (PSMA) PET scans, and how they can improve prostate cancer detection and management. The findings showed that these techniques have improved our ability to detect prostate cancer; however, further studies are needed to determine each imaging technique’s specific role in the diagnosis of prostate cancer.

**Abstract:**

Background/Objectives: Prostate cancer is a prevalent malignancy often presenting without early symptoms. Advanced imaging technologies have revolutionized its diagnosis and management. This review discusses the principles, benefits, and clinical applications of multiparametric magnetic resonance imaging (mpMRI), micro-ultrasound (microUS), and prostate-specific membrane antigen positron emission tomography–computed tomography (PSMA PET/CT) in localized prostate cancer. Methods: We conducted a comprehensive literature review of recent studies and guidelines on mpMRI, microUS, and PSMA PET/CT in prostate cancer diagnosis, focusing on their applications in biopsy-naïve patients, those with previous negative biopsies, and patients under active surveillance. Results: MpMRI has demonstrated high sensitivity and negative predictive value in detecting clinically significant prostate cancer (csPCa). MicroUS, a newer technology, has shown promising results in early studies, with sensitivity and specificity comparable to mpMRI. PSMA PET/CT has emerged as a highly sensitive and specific imaging modality, particularly valuable for staging and detecting metastatic disease. All three technologies have been incorporated into urologic practice for prostate cancer diagnosis and management, with each offering unique advantages in different clinical scenarios. Conclusions: Advanced imaging techniques, including mpMRI, microUS, and PSMA PET/CT, have significantly improved the accuracy of prostate cancer diagnosis, staging, and management. These technologies enable more precise targeting of suspicious lesions during biopsy and therapy planning. However, further research, especially randomized controlled trials, is needed to fully establish the optimal use and inclusion of these imaging modalities in various stages of prostate cancer care.

## 1. Introduction

Prostate cancer is one of the most prevalent malignancies affecting men worldwide [1]. This disease typically manifests without noticeable symptoms in its early stages and, as a result, the diagnosis and management of prostate cancer present unique challenges. It is important to accurately diagnose prostate cancers that may need treatment and those that can be more adequately followed with active surveillance [2,3]. Clinically significant prostate cancer (csPCa) is cancer that may need treatment and is commonly defined as Gleason Grade Group ≥ 2 [4,5].

Advanced imaging technologies have enhanced our ability to visualize prostate cancer. Traditional methods like transrectal ultrasound (TRUS) with standard 12 core biopsy and digital rectal examination (DRE) have limitations in terms of sensitivity and specificity, often resulting in missed or misclassified cancers [6,7]. In contrast, advanced techniques such as multiparametric magnetic resonance imaging (mpMRI), micro-ultrasound (microUS) and positron emission tomography–computed tomography (PET/CT) have emerged as improvements in prostate cancer diagnosis [8].

These advanced imaging techniques provide valuable information for staging, treatment planning, and monitoring the response to therapy. As a result, they have transformed the landscape of prostate cancer care by enabling a more tailored and precise approach to diagnosis and treatment, ultimately improving prognosis and quality of life for patients [9,10,11]. This review will discuss the principles, benefits, and clinical applications of advanced imaging in localized prostate cancer.

## 2. Multiparametric Magnetic Resonance Imaging

Magnetic resonance imaging (MRI) has fundamentally transformed the landscape of prostate cancer diagnosis and management [9,10,11]. As a non-invasive imaging technique, mpMRI offers insights into the structure and composition of the prostate gland, allowing for earlier and more accurate detection of prostate cancer [12,13]. This technology has not only revolutionized our ability to identify cancerous lesions but has also become a cornerstone in assessing the extent and aggressiveness of the disease, guiding treatment decisions, and monitoring the response to therapies.

### 2.1. Multiparametric MRI (mpMRI)

This imaging technique combines multiple imaging sequences, including T1-weighted imaging (T1WI), T2-weighted imaging (T2WI), diffusion-weighted imaging (DWI), and dynamic contrast-enhanced (DCE) imaging. Each sequence provides distinct information about the prostate and surrounding structures. T1WI provides information about regional lymph nodes and skeletal metastasis, as well as the presence of hemorrhage in the prostate [13,14,15]. T2WI provides detail about the anatomy of the prostate, and areas suspicious for prostate cancer will appear hypointense compared to normal tissue, with a lower water content and higher cellularity. T2 is the dominant factor for determining transition zone scores [13,14,15]. DWI provides information on the random motion of water molecules, with prostate cancers appearing hypointense compared to normal tissue. DWI improves the sensitivity and specificity of T2WI alone. DCE is the final sequence obtained, though it has a limited role in mpMRI, especially when diagnostic T2WI and DWI sequences are obtained; however, it is used for scoring peripheral zone lesions. When DWI or T2WI are substandard, DCE can be used to help diagnosis, though there is interest in biparametric MRI that does not include this sequence [13,16,17,18]. The combination of these data sets enhances the accuracy of cancer detection and characterization.

The Prostate Imaging Reporting and Data System (PI-RADS) is a standardized scoring system used to interpret and report the findings from multiparametric magnetic resonance imaging (mpMRI) of the prostate. PI-RADS is currently in version 2.1, which was last updated in 2019 [13]. Lesions are scored on a Likert scale from 1 to 5, with a higher number having higher suspicion for harboring csPCa. Typically, lesions given a score of PI-RADS 3 or higher will have targeted samples taken at the time of biopsy, and patients with an mpMRI PI-RADS ≤ 2 would be considered negative.

Currently, there is no consensus on the optimal number of biopsy cores needed per lesion when performing mpMRI-targeted biopsy. Biopsy protocols vary, with the number of cores taken typically ranging from one to five per lesion. In principle, the optimal number of biopsy cores taken per mpMRI lesion would minimize cost and risk to the patient, while maximizing the detection of csPCa (but not clinically insignificant prostate cancer). There are several studies that have looked at determining an optimal number of biopsy cores for an mpMRI target [19,20,21,22]. While these studies have not led to a consensus in the literature and in practice, many practitioners take on average three cores per lesion. This agrees with the current American Urological Association guideline, which recommends at least two cores per mpMRI lesion, as well as the current European Association of Urology guideline that recommend three to five targeted cores per lesion [9,10].

One method that is being assessed is the use of an In-Bore MRI-targeted prostate biopsy that can help improve targeted biopsies [23]. With most mpMRI-targeted prostate biopsies, the MRI occurs prior to the biopsy, sometimes weeks to months before. Any lesions seen on the pre-biopsy MRI are then targeted using cognitive fusion or software-guided technologies, but there is the risk of missing the lesion of interest [23]. The results from retrospective case series have shown some improvement in the detection of csPCa, though this technique requires specialized equipment and dedicated MRI time to perform [23,24,25,26,27].

Another area of interest in the diagnosis of clinically localized prostate cancer is the decision to avoid biopsy with a negative mpMRI. While there are advantages to minimizing prostate biopsies, there is a significant amount of csPCa (up to 25%) that would be missed [28,29]. As such, current guidelines recommend that a prostate biopsy can be avoided, though only in carefully selected patients, which would include those with other low-risk features such as low PSA density, low PSA, or other favorable markers that would indicate a low chance of disease [9,10,11].

mpMRI serves as a cornerstone in the comprehensive assessment of prostate cancer staging by evaluating the extent of the disease within the prostate and its adjacent structures [30,31]. In the context of local tumor extension, mpMRI excels in providing highly precise localization of the malignant lesion within the prostate gland. By discerning the extracapsular extension, mpMRI facilitates the differentiation between localized and potentially more advanced stages of prostate cancer, thereby informing treatment decisions and prognostic assessments [30,31,32]. Moreover, mpMRI’s capabilities extend to the evaluation of seminal vesicle involvement, a significant parameter in the staging process of prostate cancer [30,31].

mpMRI also plays a role in the evaluation of regional lymph nodes within the pelvic area [30]. Abnormalities or enlargement in these lymph nodes, identified through mpMRI, can be indicative of the potential dissemination of cancer beyond the prostate. While mpMRI’s capabilities in this regard are valuable for local staging and the identification of nearby lymph node involvement, the detection of distant metastases may require the application of specialized imaging modalities like bone scans, computed tomography (CT) scans, or positron emission tomography (PET) scans [9,33].

### 2.2. Biopsy-Naïve Patients

In biopsy-naïve patients, mpMRI plays a significant role in the diagnosis and staging of prostate cancer (Table 1). Prior to the widespread adoption of mpMRI, patients with a clinical suspicion of prostate cancer, determined by elevated PSA, family history, or suspicious digital rectal exam, would undergo a transrectal ultrasound-guided 12-core prostate biopsy. The standard template biopsy only sampled a small portion of the prostate and is certain to have left many patients with their cancer undetected until a further biopsy [34].

Early studies showed that the implementation of mpMRI prior to biopsy was beneficial for the detection of csPCa, with potential for improved screening and reduce rates of clinically insignificant cancer detection [40,41,42,43,44]. PROMIS in 2017 was a study of 740 biopsy-naïve men that aimed to compare the diagnostic accuracy of multiparametric MRI with that of transrectal ultrasound (TRUS) biopsy in the detection of csPCa (which they defined as Gleason score ≥ 4 + 3) [7]. The results showed that multiparametric MRI had a higher sensitivity (93% vs. 48%) and negative predictive value (89% vs. 74%) than TRUS biopsy, results that suggested that mpMRI could be used as a screening tool for csPCa [7].

Another study, MRI-FIRST, compared the diagnostic accuracy of systematic biopsy with that of targeted biopsy based on multiparametric MRI in 275 biopsy-naïve patients [36]. This study did not find any significant difference in the detection of csPCa by targeted biopsy or by systematic biopsy alone (32.3% vs. 29.9%, *p* = 0.38). If systematic biopsy was avoided, only 13 of the 94 cases of csPCa would have been missed [36].

Kasivisvanathan et al. in 2018 aimed to evaluate whether multiparametric MRI was non-inferior to standard biopsy [35]. In total, 500 biopsy-naïve patients underwent randomization in a 1:1 fashion with half undergoing standard 12-core biopsy and the remainder undergoing mpMRI, with a targeted biopsy performed only if an MRI lesion was noted. A significantly higher proportion of men in the MRI arm were diagnosed with csPCa (38% vs. 26%, *p* = 0.005), and fewer clinically insignificant prostate cancers (i.e., GG1) were diagnosed in the MRI arm by foregoing a standard biopsy for MRI-negative patients (13% less, *p* < 0.001) [35].

### 2.3. Previous Negative Biopsy

Patients with a previous negative biopsy are a special case, as they are considered lower risk than patients who are biopsy-naïve and, in many studies, are assessed independently of biopsy-naïve patients (Table 1). There is no standard for following these patients, and the decision to repeat prostate biopsy is generally based on clinical factors and increasing lab values such as PSA [9,11].

Hansen et al., in 2016, performed a multicenter prospective study of 487 patients with prior negative biopsies comparing mpMRI with saturation biopsy for the detection of csPCa [45]. MRI-targeted biopsies, in this study, missed 9% of csPCa for PI-RADS 4 and 5 lesions, and 56% for PI-RADS 3 lesions [45].

Another multi-institutional analysis by Sidana et al. looked at 779 patients who had previously had a negative systematic biopsy and high clinical suspicion of prostate cancer [37]. These patients all underwent pre-biopsy mpMRI, with a fusion targeted biopsy of MRI lesions followed by systematic biopsy. They found that fusion biopsy alone detected 205 of the 239 csPCa cases and outperformed systematic biopsy alone [37].

The current CUA guidelines recommend that for patients with prior negative biopsies, we can consider undertaking an mpMRI prior to repeat biopsy and can forego repeat biopsy if the MRI is negative, with the caveat that patients should be made aware that there is an 8–24% chance of the MRI missing csPCa [11].

### 2.4. Active Surveillance

Another area where mpMRI has taken a leading role is in patients who are managed by active surveillance (Table 1). Active surveillance is the recommended treatment for men diagnosed with low-risk prostate cancer by the AUA, EAU, and NCCN guidelines [9,33,46]. These men are followed prospectively to ensure that they do not advance to a higher-risk prostate cancer that might need treatment. Historically, these men would undergo PSA testing generally every six months, followed by random 12-core transrectal ultrasound biopsies, usually one to two years after diagnosis, and further biopsies performed when clinical suspicion of prostate cancer increased, generally due to rising PSA [2,3,34].

Ouzzane et al. found that in their study of 281 men deemed eligible for active surveillance by systematic biopsy, 28 (10%) of men were deemed ineligible based on MRI and targeted biopsy findings [38]. The ASIST trial looked at the use of mpMRI for men managed by active surveillance and randomized these 273 men to systematic biopsy or targeted biopsy alone [39]. The study found that MRI did not significantly increase the detection of csPCa in this population, with a subset determining that MRI-targeted biopsy alone did not significantly reduce the detection rate [39].

mpMRI has become part of the active surveillance protocols and is currently endorsed by the NCCN guidelines for prostate cancer [46]. Furthermore, mpMRI offers prognostic information with GG 1 cores coming from PI-RADS 3–5 lesions harboring a 3-fold increase in likelihood of later upgrading to intermediate or high-risk tumors compared to tumors found in systematic cores [47].

## 3. Micro-Ultrasound

mpMRI has become the gold standard for the diagnosis of prostate cancer, with new technologies seeking to emulate the results. mpMRI is not without fault, secondary to cost, ease of access, interuser variability, and the risk of missing clinically significant prostate cancers [48,49,50,51].

Micro-ultrasound (microUS) is a new technology developed to assist in the diagnosis of prostate cancer [51]. Unlike conventional ultrasound, microUS employs high-frequency sound waves, typically 29 MHz, compared to standard ultrasound in the 6 MHz range [52]. This high-frequency range provides significantly improved resolution, enabling the differentiation of minute structural variations within the prostate. This improved resolution is particularly valuable in detecting small tumors and differentiating between benign and malignant lesions [51]. Furthermore, microUS offers real-time imaging, which can aid in biopsy procedures and provide immediate feedback to clinicians.

**Figure 1:**
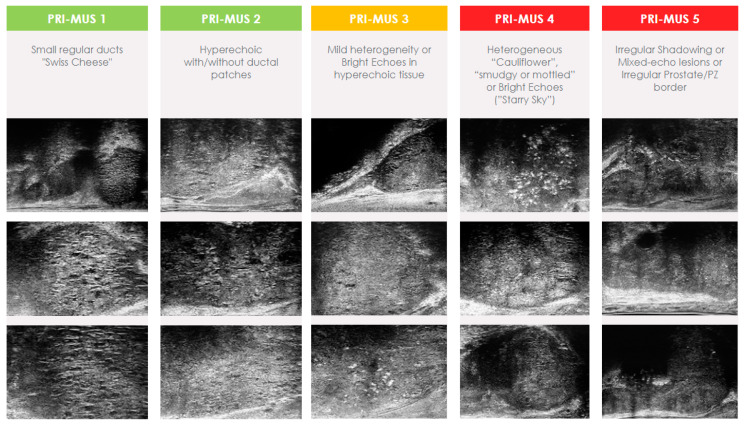
Prostate Risk Identification Using Micro-Ultrasound (PRI-MUS) score.

Much like mpMRI, microUS required the development of a standardized scoring system. The Prostate Risk Identification Using Micro-Ultrasound (PRI-MUS) standardized scoring system was developed using data from 400 microUS biopsies matched to pathology results [51]. PRI-MUS is currently in its first iteration and focuses solely on peripheral zone lesions. Lesions are assigned scores on a Likert scale ranging from 1 to 5, with a greater numerical value indicating a heightened likelihood of containing csPCa (Figure 1). Typically, lesions given a score of PRI-MUS 3 or higher will have targeted biopsy cores taken at the time of biopsy and patients with PRI-MUS ≤ 2 are considered negative.

Like MRI-targeted lesions, there is no consensus on the number of biopsy cores required per microUS lesion. Given that lesions are identified at the time of biopsy, it would be expected that fewer biopsy cores might be required, as there would be a lower risk of misregistration errors. This is an inherent drawback with MRI fusion prostate biopsies, as the MRI is not performed at the same time as the biopsy and the operator relies on imperfect software or cognitive techniques that may result in the operator missing the lesion of interest completely [53,54]. Many of the current studies in the literature do utilize three cores per target, similar to MRI, though this is not standardized [49,55,56]. The only study to date that has assessed the number of cores required for microUS-targeted biopsy by Albers et al. recommends taking three biopsy cores per sample [57].

### 3.1. Complementary Role with MRI

MicroUS is often used in conjunction with mpMRI. While MRI provides detailed anatomical information, microUS offers real-time guidance during prostate biopsy, and the benefit of most microUS platforms is that MRI lesions can be overlaid on the images for image fusion, as well [49,58].

The identification and management of anterior prostate cancer lesions pose challenges. These specific lesions, situated in the front portion of the prostate and therefore the furthest from the biopsy probe, present difficulties in both detection and sampling. Anterior prostate cancer can account for as much as 40% of prostate cancer, though it is commonly thought to be in the 15–25% range [59,60,61,62,63]. A noteworthy deficiency in the current diagnostic landscape revolves around the PRI-MUS score, which fails to incorporate the presence of anterior lesions [51]. Schaer et al. developed a scoring scheme for these anterior lesions in a two-step process, with an initial set of 102 patients used to produce their scoring scheme of “likely”, “equivocal”, or “unlikely” [50]. They then validated their results on 50 patients with microUS images who underwent radical prostatectomy, of whom 25 had confirmed anterior prostate cancer lesions and 25 did not. They found that their method was 72% sensitive and 68% specific with an ROC AUC of 0.75, indicating that microUS could ultimately be used for detecting anterior lesions [50].

### 3.2. Biopsy-Naïve Patients

Early studies found similar rates of csPCa detection when compared to mpMRI. Unfortunately, many of the early studies were retrospective in nature, not all these studies focused solely on biopsy-naïve patients, and the clinician was regularly not blinded to the mpMRI results [49,52,64,65]. These results led to a prospective registry of 11 centers that assessed 1040 men undergoing prostate biopsy with microUS after mpMRI (Table 2) [66]. Their results found that microUS was non-inferior to mpMRI for sensitivity, specificity, PPV, and NPV, and they also found that it was superior for sensitivity and NPV. While these results were promising, the biopsy was not standardized, and many of the operators were not blinded to the MRI results.

These preliminary results led to the ongoing “Optimization of Prostate Biopsy—Micro-Ultrasound versus MRI (OPTIMUM, NCT05220501)”. It is a three-arm randomized controlled trial that aims to compare microUS with mpMRI for the detection of csPCa. The planned enrollment is 1200 biopsy-naïve men, with 200 in arm 1, MicroUS only; 600 in Arm 2, mpMRI/US fusion; and the remaining 400 in arm 3, mpMRI/MicroUS Fusion (with initially blinded MRI) [55].

### 3.3. Previous Negative Biopsy

There have not been any studies to date that specifically look at this population for patients undergoing a microUS-guided prostate biopsy; however, these patients have been involved in some of the early studies, though there is not always specific information regarding their outcomes [65,69,70].

Lughezzani et al. looked at 320 patients undergoing prostate biopsy with mpMRI-detected lesion and underwent a microUS prostate biopsy with the MRI results blinded (Table 2) [67]. They did include 120 patients who had previously undergone a prostate biopsy, with this group finding overall only 22.5% csPCa compared to the biopsy-naïve with 44.5%. In the repeat biopsy cohort, microUS was able to detect all 27 csPCa [67]. To date, there have not been any randomized trials assessing solely patients with prior negative biopsy undergoing microUS prostate biopsy.

### 3.4. Active Surveillance

Many early studies did not differentiate by biopsy indication. A study by Eure et al. was among the first and looked at only nine patients, all of whom underwent an mpMRI prior to their biopsy, and compared csPCa detection by mpMRI and microUS [71]. This preliminary study showed a higher detection rate by microUS compared to MRI (89% vs. 56%, *p* = 0.02) [71].

Staerman looked at 34 consecutive patients undergoing prostate biopsy as part of their active surveillance protocol and compared mpMRI to microUS [72]. They found that csPCa detection was similar between MRI and microUS (17.6% vs. 17.6%) [72]. Another study by Maffei et al. looked at 100 prospectively enrolled patients due to undergo confirmatory biopsy one year after the diagnosis of low-risk prostate cancer (Table 2) [68]. These men all underwent an mpMRI; the results were compared to microUS and a similar sensitivity was found for the detection of csPCa (100% by MRI vs. 94.1% by microUS). Albers et al. assessed 128 consecutive patients undergoing active surveillance biopsy and found that there was no difference in the detection of csPCa by mpMRI or microUS (85% by MRI and 98% by microUS, *p* = 0.22) [58].

To date, there have not been any randomized controlled trials looking at the use of microUS in the active surveillance population. There is, however, one study ongoing by Kinnaird et al. NCT05558241—“Micro-Ultrasound In Cancer—Active Surveillance (MUSIC-AS)”. This is a multicenter, paired diagnostic trial of 210 men, comparing microUS to mpMRI for the detection of csPCa for men on active surveillance undergoing their confirmatory biopsy. All men will receive a pre-biopsy mpMRI and undergo a microUS-guided biopsy; the operator is blinded to the MRI results until after they have assessed the prostate for any microUS lesions.

## 4. PSMA PET

Prostate-specific membrane antigen positron emission tomography (PSMA PET) scan is a cutting-edge imaging technique for the diagnosis and management of prostate cancer [73,74]. It is particularly valuable in detecting and localizing prostate cancer, both in its early stages and when it has metastasized. The normal in vivo biodistribution of PSMA on imaging is seen in the liver, spleen, bowel, parotid, lacrimal glands, and salivary glands, as well as the kidneys, ureter, and bladder [75]. In the majority of prostate cancers, the expression of PSMA is significantly increased, making it an ideal target for imaging [76]. PET scans are commonly combined with computed tomography (PET/CT) or with MRI (PET/MRI) [77]. PSMA-PET scans involve the use of a radiotracer that binds to the PSMA protein, making these scans highly sensitive and specific in the detection of prostate cancer [73,74].

There are currently two main types of PSMA PET radioligands, those that use Gallium-68 as their radioisotope and those that use Fluorine-18, each with different sets of benefits and challenges [76,78,79]. Gallium-68 radioligands were among the first utilized; however, there are several shortcomings, including a shorter half-life, kidney-dominant excretion, and lower resolution than fluorine equivalents [76,78,79].

While PSMA is currently the major molecular imaging target for prostate cancer, it is not the sole target that is being investigated [80,81,82]. ^18^F-Choline and ^18^F-Fluciclovine are among two other radiopharmaceutical agents, though both are non-specific to prostate cancer. Choline is necessary for cell membranes, and tumors with high proliferation will show an increased uptake [83]. Fluciclovine is an amino acid analog, and in prostate cancer, amino acid transporters are upregulated, increasing the transport of this agent into cells with prostate cancer [84,85]. Choline uptake can be seen in BPH, lymphomas, renal cell carcinoma (RCC), and thymomas, and Fluciclovine uptake can be seen in several other types of cancer, including RCC, oropharyngeal squamous cell carcinomas, meningiomas, breast cancers, lung cancers and colorectal cancers [80,86]. Another target being investigated is the Gastrin-releasing peptide receptor (GRPR) [82]. GRPR is upregulated in many types of cancers, including prostate, breast, lung, and colorectal cancers, and can potentially detect PSMA-negative prostate cancers [82]. GRPR has also been shown to have elevated expression in lower-grade prostate cancers [82,87,88,89].

There are several standardized scoring systems for the interpretation of PSMA-targeted PET imaging but, unlike mpMRI and micro-ultrasound, there is not a single widely accepted scoring system. Standardized scoring systems such as the PROMISE v2 score or the PSMA-Rads score, an miTNM score, aim to harmonize interpretation across readers and institutions [90,91,92,93,94]. The PROMISE v2 framework is of particular note and has seen widespread use and adoption for its versatility [94]. This framework allows the staging of localized disease and has adopted the 5-point PRIMARY score for grading intraprostatic lesions. A PRIMARY score of 1–2 would be considered negative, and scores of 3–5 would be considered positive, with an associated T stage based on the presumed extent seen on PSMA-PET/CT.

PSMA PET/CT scans play a role in staging patients with recurrent or metastatic prostate cancer and have the added benefit of a whole-body assessment that mpMRI and micro-ultrasound do not provide. They excel at locating metastatic lesions, even in cases involving small-volume pelvic lymph nodes that may not meet size criteria on conventional imaging or those situated in atypical locations, such as the adrenal glands, penis, and brain [95,96,97]. Additionally, PSMA PET/CT scans are valuable for monitoring for recurrence after treatment [98].

While PSMA PET/CT is more common, PSMA PET can be combined with MRI. When assessing localized disease, PSMA-PET/MRI appears to offer improved detection of prostate cancer when compared to PSMA-PET/CT, due to MRI’s ability to better differentiate pelvic structures compared to CT [99,100]. Using MRI instead of CT should improve the visualization of pelvic structures due to the higher soft tissue contrast [101]. A recent meta-analysis found that PSMA-PET/CT and PSMA-PET/MRI had comparable rates of detection of recurrent PCa, though with low patient enrollment [101]. PSMA-PET/MRI is also beneficial for local lymph node disease and shows similar results to PSMA-PET/CT for visceral and bony metastases [99]. Many studies utilize PSMA PET/CT instead of PSMA PET/MRI due to the relatively low cost, faster scan, and availability of scanners [99,100].

Furthermore, several studies have shown the safety and efficacy of PSMA-targeted radioligand therapy, highlighting its use as a theranostic agent. Clinical trials have shown the efficacy of PSMA-targeted therapies, such as ^177^Lu-PSMA-617, showing favorable results in improving patient outcomes in metastatic castration-resistant prostate cancer [102]. The VISION trial was an international multicentered trial that showed that the addition of ^177^Lu-PSMA-617 in mCRPC previously treated with at least one taxane regime and at least one androgen-receptor-pathway inhibitor had significantly improved progression-free survival and overall survival when compared to those treated with the standard of care [103]. With these successes, PSMA-targeted radioligand therapies are being assessed in earlier portions of the treatment sequence for metastatic prostate cancer. This is seen in the PSMAfore study, which assessed the use of ^177^Lu-PSMA-617 in taxane-naïve patients with PSMA-positive mCRPC who had progressed on previous androgen receptor pathway inhibitor (ARPI). They found that when compared to a change in ARPI, ^177^Lu-PSMA-617 had prolonged radiographic progression-free survival compared to ARPI and should be considered as an alternative treatment [104].

### 4.1. Biopsy-Naïve Patients

Despite their early role mainly representing a test to assess for nodal or metastatic spread, PSMA PET/CT scans have recently received increased attention regarding the utilization of the test for primary diagnosis and biopsy targeting (Table 3). Similarly to microUS, many of the early PSMA PET/CT studies did not solely look at biopsy-naïve patients. However, one such study by Pepe et al. compared PSMA PET/CT to the current standard mpMRI and found improved rates of detection for csPCa by PSMA PET/CT and mpMRI (95.4% vs. 81.8%), though it is unclear in this study whether the clinician was blinded to MRI results at the time of biopsy [105]. This study, among others, prompted researchers to design randomized trials, aiming to rigorously assess the clinical utility and impact of PSMA PET/CT in larger cohorts of biopsy-naïve patients.

PRIMARY by Emmett et al. was a prospective multicenter phase II imaging trial that aimed to determine whether the combination of 68-Ga-PSMA-11-PET/CT and mpMRI was superior to mpMRI alone in the diagnostic performance for detecting csPCa [91]. The study enrolled 296 men with suspected prostate cancer, and the results indicated that the combination of mpMRI and PSMA PET/CT reduced false negatives for csPCa compared with MRI alone, potentially allowing a reduction in the number of prostate biopsies required to diagnose csPCa [91].

Another ongoing trial is the DEPROMP Trial that is looking at the addition of PSMA PET/CT scans to the current standard of care (mpMRI-guided systematic and targeted biopsy) [108]. This trial seeks to compare the current gold-standard mpMRI-guided prostate biopsy to PSMA-PET/CT-guided prostate biopsy and compare the impact that PSMA PET scans have on the treatment plan. Interim results from the DEPROMP trial including the first 100 patients found that the addition of PSMA-PET/CT to mpMRI-guided prostate biopsy increased the detection of csPCa by 4% and found a 2% decrease in non-clinically significant prostate cancer. When comparing PSMA-PET/CT-guided biopsy with MRI-guided biopsy, the authors found that PSMA-PET/CT-guided biopsy led to a 3% increase in csPCa detection and a 1% decrease in the detection of non-clinically significant prostate cancer and was non-inferior to mpMRI [111].

**Figure 2:**
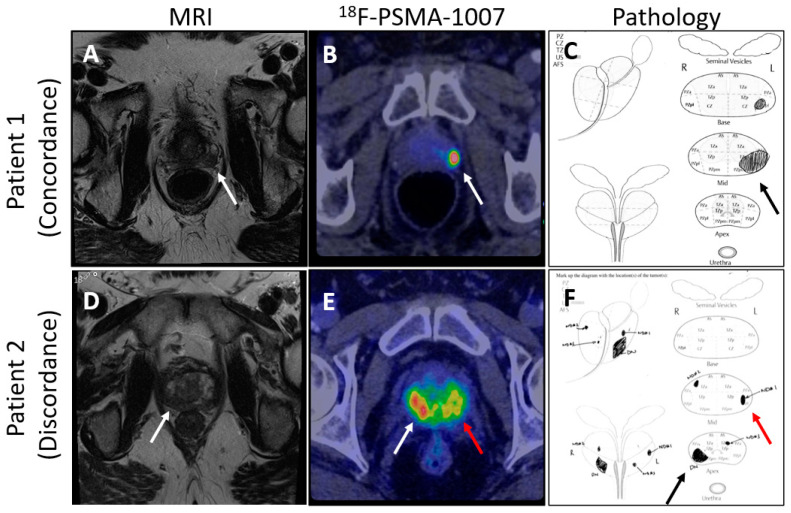
Comparison of mpMRI and PSMA-PET images showing concordance and discordance between the imaging techniques and pathology. (Arrows point to areas of suspected prostate cancer on imaging and confirmed diagnosis on pathology; (**A**) shows an MRI lesion on patient 1’s left mid gland, (**B**) shows suspected prostate cancer by ^18^F-PSMA-1007 in patient 1’s left mid gland, (**C**) shows prostate cancer found by pathology review in the left mid gland of the prostate, (**D**) shows an MRI lesion on patient 2’s right apex, (**E**) shows suspected bilateral prostate cancer by ^18^F-PSMA-1007 in patient 2, (**F**) shows bilateral prostate cancer found on pathology review of the specimen).

The Next Generation trial by Mookerji et al. compared the diagnostic accuracy of 18F-PSMA-1007 PET/CT and multiparametric MRI (mpMRI) for the locoregional staging of intermediate-risk and high-risk prostate cancer [112]. Conducted as a phase 2 prospective validating paired cohort study, it involved 134 men who underwent both imaging modalities before radical prostatectomy. The findings demonstrated that 18F-PSMA-1007 PET/CT was significantly more accurate than mpMRI in correctly identifying the final pathological tumor stage, dominant nodule, laterality, and extracapsular extension, though not in seminal vesicle invasion (Figure 2). These results support the use of 18F-PSMA-1007 PET/CT in the preoperative staging of prostate cancer, suggesting that it may offer superior accuracy over mpMRI [112].

### 4.2. Previous Negative Biopsy

PSMA PET/CT seeks to improve on conventional diagnostic tests and imaging in the special subset of patients that have already had a negative prostate biopsy and, at times, a negative mpMRI and who continue to have a high clinical suspicion of prostate cancer. A study by Lopci et al. prospectively enrolled 45 patients, all of whom had previously had at least one negative prostate biopsy with persistently elevated PSA and clinical suspicion of prostate cancer [106]. These men had previously undergone an mpMRI that was negative or had contraindications to MRI. Of the 45 patients, 25 had a positive 68-GA-PSMA PET/CT scan, with 7 of these 25 patients found to have csPCa in this very select patient population [106].

Liu et al. looked at 31 patients with a previously negative prostate biopsy but persistent elevated serum prostate-specific antigen (PSA) [113]. These men all underwent a 68-GA-PSMA-11 PET/CT scan and underwent a standard systematic biopsy with PET-guided biopsy cores for any PET-suspicious lesions. This study did not compare with the current gold standard of mpMRI-guided biopsy; however, the authors did find that PSMA PET/CT could be a useful adjunct for the detection of csPCa in the previous-negative-biopsy population [113].

To date, there are no randomized control trials for this patient population; however, with the rise in interest in this technology, there will likely be several further studies assessing the benefit of this technology. There is currently at least one study enrolling this patient population—PROSPET-BX [109]. This study is a paired diagnostic superiority trial comparing PSMA-PET/CT-guided targeted prostate biopsy against mpMRI-guided targeted prostate biopsy to determine whether PSMA-PET/CT has a superior diagnostic performance to MRI. Their interim analysis found that the addition of PSMA PET/CT to MRI results detected 90% of csPCa [114].

### 4.3. Active Surveillance

This is an area where PSMA PET/CT scans seek to improve the detection of intermediate and high-risk cancers while avoiding biopsies and minimizing the detection of clinically insignificant or low-risk prostate cancers. In recent years, there have been several studies that have assessed this patient population. Pepe et al. assessed 40 men followed by active surveillance due for prostate biopsy who underwent an mpMRI and PSMA PET/CT scan [107]. This study only found three patients with csPCa, with a 66% detection rate by both MRI and PSMA PET/CT.

Another feasibility study was performed by Jain et al., assessing the use of PSMA PET/CT scans after the diagnosis of low-risk prostate cancer [115]. This study looked at 30 men diagnosed with low- or favorable-intermediate-risk prostate cancer who underwent a PSMA PET/CT after diagnosis. Of these 30 men, 19 went on to undergo treatment, with 15 of these cases being because of concerning features on PSMA PET/CT [115].

To date, there are no randomized controlled trials, and the literature is minimal, regarding PSMA PET/CT in the active surveillance population for primary diagnosis. There is an ongoing prospective cross-sectional partially blinded multicenter clinical trial assessing the added value of PSMA PET/CT in addition to mpMRI in men undergoing biopsy during active surveillance for low- to intermediate-risk prostate cancer [110]. This trial seeks to enroll 225 men and compare the diagnostic capability of PSMA PET/CT and mpMRI in the active surveillance population. While there is not enough evidence to support the use of PSMA PET/CT as the sole screening test in active surveillance patients, it is possible that PSMA PET/CT may be able to prevent biopsies and more accurately detect prostate cancer than conventional methods in the future.

## 5. Conclusions

The primary diagnosis of prostate cancer has witnessed remarkable advancements with the integration of cutting-edge imaging modalities such as mpMRI, micro-ultrasound, and PSMA PET. Multiparametric magnetic resonance imaging (mpMRI) has emerged as a powerful tool for prostate cancer detection, offering high resolution and detailed anatomical information. Micro-ultrasound, with its enhanced imaging capabilities at the microscopic level, provides additional precision in identifying suspicious lesions, with associated high sensitivity for clinically significant prostate cancer. Furthermore, the inclusion of PSMA PET imaging has significantly improved sensitivity and specificity, enabling clinicians to detect lesions with higher accuracy and delineate the extent of disease more effectively. The synergistic use of these imaging technologies not only enhances diagnostic accuracy but also aids in personalized treatment planning, ultimately contributing to improved patient outcomes in the management of prostate cancer.

## Figures and Tables

**Table 1:** Major MRI studies.

Study	Design	Population	Key Findings
Ahmed et al. (2017)—PROMIS [7]	Paired-cohort confirmatory test comparing MP-MRI against TRUS-biopsy with template biopsy as reference	Biopsy-naïve	Multiparametric MRI (mpMRI) had a sensitivity of 93% for detecting clinically significant prostate cancer (Gleason score ≥ 3 + 4), compared to 48% with standard TRUS-guided biopsy. The negative predictive value of mpMRI was 89%. Additionally, using mpMRI could have spared 27% of men from undergoing a biopsy.
Kasivisvanathan et al. (2018)—PRECISION [35]	Randomized non-inferiority trial comparing MRI-targeted biopsies against standard 12-core biopsy	Biopsy-naïve	In the mpMRI-targeted biopsy group, 38% of men were diagnosed with clinically significant prostate cancer (Gleason score ≥ 3 + 4) compared to 26% in the standard TRUS-guided biopsy group. The number of men diagnosed with clinically insignificant prostate cancer (Gleason score ≤ 3 + 3) was lower in the mpMRI group (9%) compared to the TRUS group (22%). The study also showed that 28% of men could avoid a biopsy entirely if the mpMRI did not show any suspicious lesions.
Rouvière et al. (2019)—MRI First [36]	Paired diagnostic study comparing mpMRI-targeted biopsies against standard biopsy	Biopsy-naïve	mpMRI followed by targeted biopsy detected 51% of clinically significant prostate cancers (Gleason score ≥ 3 + 4) compared to 37% detected by systematic biopsy alone. The study also noted that 23% of men could avoid a biopsy if the mpMRI showed no suspicious lesions, and the approach led to fewer diagnoses of clinically insignificant cancers (4% with MRI-targeted biopsy vs. 12% with systematic biopsy).
Sidana et al. (2018) [37]	Retrospective multicentre analysis comparing targeted and systematic biopsies	Prior negative biopsies	Targeted prostate biopsy detected clinically significant cancer in 26.3% of patients, whereas 12-core systematic biopsy detected it in 18.8% of patients, with systematic biopsy diagnosing only an additional 4.4%.
Ouzzane et al. (2015) [38]	Prospective multicenter study comparing systematic biopsies to mpMRI targeted biopsy	Active surveillance	mpMRI-targeted biopsies found that 10% of men deemed eligible for active surveillance with positive mpMRI were found to have clinically significant prostate cancer on targeted biopsy specimens.
Klotz et al. (2019)—ASIST [39]	Randomized trial comparing systematic biopsy with systematic and mpMRI targeted biopsy	Active surveillance	mpMRI-targeted biopsy did not significantly improve the clinically significant prostate cancer detection rate (mpMRI-targeted 21.2% vs. systematic 22.8% ≥ GG2).

**Table 2:** Major micro-ultrasound studies.

Study	Design	Population	Key Findings
Klotz et al. (2020) [66]	Multicenter, prospective registry comparing mpMRI and microUS	1040 patients, 352 with prior biopsy, with 286 prior negative	microUS found to have higher sensitivity compared to mpMRI (94% vs. 90%, *p* = 0.03) and NPV (85% vs. 77%, *p* = 0.04) for the detection of clinically significant prostate cancer (≥GG2)
Lughezzani et al. (2021) [67]	Single-institution cohort study	320 patients, with 200 biopsy-naïve	microUS found to have a sensitivity of 89.7% and NPV of 81.5% for the detection of csPCa, with a higher sensitivity and NPV for patients undergoing repeat biopsy
Albers et al. (2022) [58]	Single-institution cohort study comparing microUS and mpMRI	Active surveillance	microUS found to have a sensitivity of 97% and NPV of 97% with an mpMRI sensitivity of 85% and NPV of 91% for the detection of csPCa
Maffei et al. (2023) [68]	Single-institution cohort study comparing microUS and mpMRI	Active surveillance—confirmatory biopsy	microUS found to have 94.1% sensitivity and NPV of 88.9% with an mpMRI sensitivity of 100% and NPV of 100% for the detection of csPCa
Klotz et al.—OPTIMUM [55]	Three-arm multicentered randomized controlled trial, comparing mpMRI/microUS, microUS only, and mpMRI/US fusion biopsy	Biopsy-naïve	Trial ongoing, planned enrollment of 1200 men with interim analysis planned at 600 patients. NCT05220501
Kinnaird et al.—MUSIC-AS	Multicenter paired diagnostic trial comparing mpMRI and microUS	Active surveillance	Trial ongoing, planned enrollment of 210 men. NCT05558241

**Table 3:** Major PSMA PET studies.

Study	Design	Population	Key Findings
Pepe et al. (2022) [105]	Single-institution cohort study comparing ^68^Ga-PSMA-11/CT and mpMRI	Mix of biopsy-naïve and repeat biopsy	PSMA PET/CT-targeted biopsy had a sensitivity of 95.4% compared to MRI with 81.8% for the detection of csPCa. PSMA PET had an NPV of 96.5% compared with 87.5% for MRI.
Lopci et al. (2018) [106]	Single-institution prospective observational study assessing ^68^Ga-PSMA	Prior negative biopsy	Of the 45 men enrolled, 25 had positive PSMA PET/CT scans, with csPCa found in 7 men.
Pepe et al. (2023) [107]	Single-institution cohort study comparing ^68^Ga-PSMA-11 PET/CT and mpMRI	Active surveillance	Only three men found to have csPCa, with MRI detecting 66% and PSMA PET/CT also 66%.
Emmett et al. (2021)—PRIMARY [91]	Prospective, multicentered, paired diagnostic trial of ^68^Ga-PSMA-11 PET/CT and mpMRI	Biopsy-naïve	Combination of PSMA PET/CT + MRI improved NPV from 72% for MRI alone to 91% (*p* < 0.001) for the detection of csPCa. The sensitivity with the addition of PSMA PET/CT improved from 83% to 97% (*p* < 0.001), with an associated reduction in specificity from 53% to 40% (*p* = 0.011).
Krausewitz et al.—DEPROMP [108]	Prospective trial assessing the benefit of adding ^68^Ga-PSMA-11 PET/CT to standard-of-care mpMRI-guided prostate biopsy	Biopsy-naïve	Trial ongoing, with planned recruitment of 230 men. Interim analysis found the addition of PSMA-PET/CT to MRI results increased csPCa detection by 3% and PSMA-PET/CT was non-inferior to mpMRI for the detection of csPCa when used alone.
Lughezzani et al.—PROSPET-BX [109]	Prospective trial assessing the benefit of adding ^68^Ga-PSMA-11PET/CT to standard-of-care mpMRI-guided prostate biopsy	Prior negative biopsy	Trial ongoing, with planned recruitment of 128 men. (NCT05297162) Interim analysis found the addition of PSMA PET/CT to MRI results detected 90% of csPCa.
Thompson et al. [110]	Prospective multicentered trial assessing ^68^Ga-PSMA-11 PET/CT in addition to mpMRI	Active surveillance	Trial ongoing, with planned recruitment of 225 men. (ACTRN12622000188730)

## Data Availability

All publications can be found on PubMed.

## References

[1] Bray F., Laversanne M., Sung H., Ferlay J., Siegel R.L., Soerjomataram I., Jemal A. (2024). Global Cancer Statistics 2022: GLOBOCAN Estimates of Incidence and Mortality Worldwide for 36 Cancers in 185 Countries. CA Cancer J. Clin..

[2] Tosoian J.J., Mamawala M., Epstein J.I., Landis P., Macura K.J., Simopoulos D.N., Carter H.B., Gorin M.A. (2020). Active Surveillance of Grade Group 1 Prostate Cancer: Long-Term Outcomes from a Large Prospective Cohort. Eur. Urol..

[3] Litwin M.S., Tan H.J. (2017). The Diagnosis and Treatment of Prostate Cancer: A Review. JAMA J. Am. Med. Assoc..

[4] Cowan T., Baker E., McCray G., Reeves F., Houlihan K., Johns-Putra L. (2020). Detection of Clinically Significant Cancer in the Anterior Prostate by Transperineal Biopsy. BJU Int..

[5] Matoso A., Epstein J.I. (2019). Defining Clinically Significant Prostate Cancer on the Basis of Pathological Findings. Histopathology.

[6] Corcoran N.M., Hong M.K.H., Casey R.G., Hurtado-Coll A., Peters J., Harewood L., Goldenberg S.L., Hovens C.M., Costello A.J., Gleave M.E. (2011). Upgrade in Gleason Score between Prostate Biopsies and Pathology Following Radical Prostatectomy Significantly Impacts upon the Risk of Biochemical Recurrence. BJU Int..

[7] Ahmed H.U., El-Shater Bosaily A., Brown L.C., Gabe R., Kaplan R., Parmar M.K., Collaco-Moraes Y., Ward K., Hindley R.G., Freeman A. (2017). Diagnostic Accuracy of Multi-Parametric MRI and TRUS Biopsy in Prostate Cancer (PROMIS): A Paired Validating Confirmatory Study. Lancet.

[8] Dos Santos D.N., Horvat N., Dias A.B., Mota M., Filho G., Schoen K., Ghai S., Queiroz M., Viana P. (2022). Prostate Cancer Imaging: What We Already Know and What Is on the Horizon. Radiographics.

[9] EAU Guidelines. https://uroweb.org/guidelines/prostate-cancer/chapter/diagnostic-evaluation.

[10] Wei J.T., Barocas D., Carlsson S., Coakley F., Eggener S., Etzioni R., Fine S.W., Han M., Konety B.R., Miner M. (2023). Early Detection of Prostate Cancer: AUA/SUO Guideline Part II: Considerations for a Prostate Biopsy. J. Urol..

[11] Mason R.J., Marzouk K., Finelli A., Saad F., So A.I., Violette P.D., Breau R.H., Rendon R.A. (2022). UPDATE—2022 Canadian Urological Association Recommendations on Prostate Cancer Screening and Early Diagnosis: Endorsement of the 2021 Cancer Care Ontario Guidelines on Prostate Multiparametric Magnetic Resonance Imaging. Yosetsu Gakkai Shi/J. Jpn. Weld. Soc..

[12] Giganti F., Rosenkrantz A.B., Villeirs G., Panebianco V., Stabile A., Emberton M., Moore C.M. (2019). The Evolution of MRI of the Prostate: The Past, the Present, and the Future. Am. J. Roentgenol..

[13] Turkbey B., Rosenkrantz A.B., Haider M.A., Padhani A.R., Villeirs G., Macura K.J., Tempany C.M., Choyke P.L., Cornud F., Margolis D.J. (2019). Prostate Imaging Reporting and Data System Version 2.1: 2019 Update of Prostate Imaging Reporting and Data System Version 2. Eur. Urol..

[14] Demirel H.C., Davis J.W. (2018). Multiparametric Magnetic Resonance Imaging: Overview of the Technique, Clinical Applications in Prostate Biopsy and Future Directions. Turk. J. Urol..

[15] Weinreb J.C., Barentsz J.O., Choyke P.L., Cornud F., Haider M.A., Macura K.J., Margolis D., Schnall M.D., Shtern F., Tempany C.M. (2016). PI-RADS Prostate Imaging—Reporting and Data System: 2015, Version 2. Eur. Urol..

[16] Belue M.J., Yilmaz E.C., Daryanani A., Turkbey B. (2022). Current Status of Biparametric MRI in Prostate Cancer Diagnosis: Literature Analysis. Life.

[17] Iacob R., Stoicescu E.R., Cerbu S., Manolescu D.L., Bardan R., Cumpănaş A. (2023). Could Biparametric MRI Replace Multiparametric MRI in the Management of Prostate Cancer?. Life.

[18] Asif A., Nathan A., Ng A., Khetrapal P., Chan V.W.S., Giganti F., Allen C., Freeman A., Punwani S., Lorgelly P. (2023). Comparing Biparametric to Multiparametric MRI in the Diagnosis of Clinically Significant Prostate Cancer in Biopsy-Naive Men (PRIME): A Prospective, International, Multicentre, Non-Inferiority within-Patient, Diagnostic Yield Trial Protocol. BMJ Open.

[19] Zhang M., Milot L., Khalvati F., Sugar L., Downes M., Baig S.M., Klotz L., Haider M.A. (2019). Value of Increasing Biopsy Cores per Target with Cognitive MRI-Targeted Transrectal US Prostate Biopsy. Radiology.

[20] Dimitroulis P., Rabenalt R., Nini A., Hiester A., Esposito I., Schimmöller L., Antoch G., Albers P., Arsov C. (2018). Multiparametric Magnetic Resonance Imaging/Ultrasound Fusion Prostate Biopsy—Are 2 Biopsy Cores per Magnetic Resonance Imaging Lesion Required?. J. Urol..

[21] Cetin S., Huseyinli A., Koparal M.Y., Bulut E.C., Ucar M., Gonul I.I., Sozen S. (2023). How Many Cores Should Be Taken from Each Region of Interest When Performing a Targeted Transrectal Prostate Biopsy?. Prostate Int..

[22] Subramanian N., Recchimuzzi D.Z., Xi Y., Diaz De Leon A., Chen H., Xie D., Goldberg K., Rofsky N.M., Pedrosa I., Costa D.N. (2021). Impact of the Number of Cores on the Prostate Cancer Detection Rate in Men Undergoing In-Bore Magnetic Resonance Imaging-Guided Targeted Biopsies. J. Comput. Assist. Tomogr..

[23] Recchimuzzi D.Z., de Leon A.D., Pedrosa I., Travalini D., Latin H., Goldberg K., Meng X., Begovic J., Rayan J., Roehrborn C.G. (2024). Direct MRI-Guided In-Bore Targeted Biopsy of the Prostate: A Step-by-Step How To and Lessons Learned. Radiographics.

[24] Costa D.N., Goldberg K., de Leon A.D., Lotan Y., Xi Y., Aziz M., Freifeld Y., Margulis V., Raj G., Roehrborn C.G. (2019). Magnetic Resonance Imaging–Guided In-Bore and Magnetic Resonance Imaging-Transrectal Ultrasound Fusion Targeted Prostate Biopsies: An Adjusted Comparison of Clinically Significant Prostate Cancer Detection Rate. Eur. Urol. Oncol..

[25] Venderink W., van der Leest M., van Luijtelaar A., van de Ven W.J.M., Fütterer J.J., Sedelaar J.P.M., Huisman H.J. (2017). Retrospective Comparison of Direct In-Bore Magnetic Resonance Imaging (MRI)-Guided Biopsy and Fusion-Guided Biopsy in Patients with MRI Lesions Which Are Likely or Highly Likely to Be Clinically Significant Prostate Cancer. World J. Urol..

[26] Ramos F., Korets R., Fleishman A., Kaul S., Johnson M., Wei J.L., Olumi A.F., Tsai L.L., Gershman B. (2023). Comparative Effectiveness of Magnetic Resonance Imaging-Ultrasound Fusion Versus In-Bore Magnetic Resonance Imaging-Targeted Prostate Biopsy. Urology.

[27] Arsov C., Rabenalt R., Blondin D., Quentin M., Hiester A., Godehardt E., Gabbert H.E., Becker N., Antoch G., Albers P. (2015). Prospective Randomized Trial Comparing Magnetic Resonance Imaging (MRI)-Guided In-Bore Biopsy to MRI-Ultrasound Fusion and Transrectal Ultrasound-Guided Prostate Biopsy in Patients with Prior Negative Biopsies. Eur. Urol..

[28] Lovegrove C.E., Miah S., El-Shater Bosaily A., Bott S., Brown L., Burns-Cox N., Dudderidge T., Freeman A., Henderson A., Hindley R. (2020). Comparison of Transrectal Ultrasound Biopsy to Transperineal Template Mapping Biopsies Stratified by Multiparametric Magnetic Resonance Imaging Score in the PROMIS Trial. J. Urol..

[29] Moldovan P.C., Van den Broeck T., Sylvester R., Marconi L., Bellmunt J., van den Bergh R.C.N., Bolla M., Briers E., Cumberbatch M.G., Fossati N. (2017). What Is the Negative Predictive Value of Multiparametric Magnetic Resonance Imaging in Excluding Prostate Cancer at Biopsy? A Systematic Review and Meta-Analysis from the European Association of Urology Prostate Cancer Guidelines Panel. Eur. Urol..

[30] Caglic I., Kovac V., Barrett T. (2019). Multiparametric MRI—Local Staging of Prostate Cancer and Beyond. Radiol. Oncol..

[31] de Rooij M., Hamoen E.H.J., Witjes J.A., Barentsz J.O., Rovers M.M. (2016). Accuracy of Magnetic Resonance Imaging for Local Staging of Prostate Cancer: A Diagnostic Meta-Analysis. Eur. Urol..

[32] Sebesta E.M., Anderson C.B. (2017). The Surgical Management of Prostate Cancer. Semin. Oncol..

[33] Eastham J.A., Auffenberg G.B., Barocas D.A., Chou R., Crispino T., Davis J.W., Eggener S., Horwitz E.M., Kane C.J., Lin D.W. (2022). Clinically Localized Prostate Cancer: AUA/ASTRO Guideline 2022. J. Urol..

[34] Klotz L., Vesprini D., Sethukavalan P., Jethava V., Zhang L., Jain S., Yamamoto T., Mamedov A., Loblaw A. (2015). Long-Term Follow-up of a Large Active Surveillance Cohort of Patients with Prostate Cancer. J. Clin. Oncol..

[35] Kasivisvanathan V., Rannikko A.S., Borghi M., Panebianco V., Mynderse L.A., Vaarala M.H., Briganti A., Budäus L., Hellawell G., Hindley R.G. (2018). MRI-Targeted or Standard Biopsy for Prostate-Cancer Diagnosis. N. Engl. J. Med..

[36] Rouvière O., Puech P., Renard-Penna R., Claudon M., Roy C., Mège-Lechevallier F., Decaussin-Petrucci M., Dubreuil-Chambardel M., Magaud L., Remontet L. (2019). Use of Prostate Systematic and Targeted Biopsy on the Basis of Multiparametric MRI in Biopsy-Naive Patients (MRI-FIRST): A Prospective, Multicentre, Paired Diagnostic Study. Lancet Oncol..

[37] Sidana A., Watson M.J., George A.K., Rastinehad A.R., Vourganti S., Rais-Bahrami S., Muthigi A., Maruf M., Gordetsky J.B., Nix J.W. (2018). Fusion Prostate Biopsy Outperforms 12-Core Systematic Prostate Biopsy in Patients with Prior Negative Systematic Biopsy: A Multi-Institutional Analysis. Urol. Oncol..

[38] Ouzzane A., Renard-Penna R., Marliere F., Mozer P., Olivier J., Barkatz J., Puech P., Villers A. (2015). Magnetic Resonance Imaging Targeted Biopsy Improves Selection of Patients Considered for Active Surveillance for Clinically Low Risk Prostate Cancer Based on Systematic Biopsies. J. Urol..

[39] Klotz L., Loblaw A., Sugar L., Moussa M., Berman D.M., Van der Kwast T., Vesprini D., Milot L., Kebabdjian M., Fleshner N. (2019). Active Surveillance Magnetic Resonance Imaging Study (ASIST): Results of a Randomized Multicenter Prospective Trial. Eur. Urol..

[40] Turkbey B., Brown A.M., Sankineni S., Wood B.J., Pinto P.A., Choyke P.L. (2016). Multiparametric Prostate Magnetic Resonance Imaging in the Evaluation of Prostate Cancer. CA Cancer J. Clin..

[41] Mowatt G., Scotland G., Boachie C., Cruickshank M., Ford J.A., Fraser C., Kurban L., Lam T.B., Padhani A.R., Royle J. (2013). The Diagnostic Accuracy and Cost-Effectiveness of Magnetic Resonance Spectroscopy and Enhanced Magnetic Resonance Imaging Techniques in Aiding the Localisation of Prostate Abnormalities for Biopsy: A Systematic Review and Economic Evaluation. Health Technol. Assess..

[42] Valerio M., Willis S., Van Der Meulen J., Emberton M., Ahmed H.U. (2015). Methodological Considerations in Assessing the Utility of Imaging in Early Prostate Cancer. Curr. Opin. Urol..

[43] Ahmed H.U., Kirkham A., Arya M., Illing R., Freeman A., Allen C., Emberton M. (2009). Is It Time to Consider a Role for MRI before Prostate Biopsy?. Nat. Rev. Clin. Oncol..

[44] Siddiqui M.M., Rais-Bahrami S., Turkbey B., George A.K., Rothwax J., Shakir N., Okoro C., Raskolnikov D., Parnes H.L., Linehan W.M. (2015). Comparison of MR/Ultrasound Fusion–Guided Biopsy With Ultrasound-Guided Biopsy for the Diagnosis of Prostate Cancer. JAMA.

[45] Hansen N.L., Kesch C., Barrett T., Koo B., Radtke J.P., Bonekamp D., Schlemmer H.P., Warren A.Y., Wieczorek K., Hohenfellner M. (2017). Multicentre Evaluation of Targeted and Systematic Biopsies Using Magnetic Resonance and Ultrasound Image-Fusion Guided Transperineal Prostate Biopsy in Patients with a Previous Negative Biopsy. BJU Int..

[46] Schaeffer E.M., Srinivas S., Adra N., An Y., Barocas D., Bitting R., Bryce A., Chapin B., Cheng H.H., D’Amico A.V. (2023). Prostate Cancer, Version 4.2023, NCCN Clinical Practice Guidelines in Oncology. J. Natl. Compr. Cancer Netw..

[47] Kinnaird A., Yerram N.K., O’Connor L., Brisbane W., Sharma V., Chuang R., Jayadevan R., Ahdoot M., Daneshvar M., Priester A. (2022). Magnetic Resonance Imaging-Guided Biopsy in Active Surveillance of Prostate Cancer. J. Urol..

[48] Fusco F., Emberton M., Arcaniolo D., De Nunzio C., Manfredi C., Creta M. (2022). Prostatic High-Resolution Micro-Ultrasound: An Attractive Step-Forward in the Management of Prostate Cancer Patients. Prostate Cancer Prostatic Dis..

[49] Wang B., Broomfield S., Medina Martín A., Albers P., Fung C., Kinnaird A. (2023). Detection of Clinically Significant Prostate Cancer by Micro-Ultrasound-Informed Systematic Biopsy during MRI/Micro-Ultrasound Fusion Biopsy. Can. Urol. Assoc. J..

[50] Schaer S., Rakauskas A., Dagher J., La Rosa S., Pensa J., Brisbane W., Marks L., Kinnaird A., Abouassaly R., Klein E. (2023). Assessing Cancer Risk in the Anterior Part of the Prostate Using Micro-Ultrasound: Validation of a Novel Distinct Protocol. World J. Urol..

[51] Ghai S., Eure G., Fradet V., Hyndman M.E., McGrath T., Wodlinger B., Pavlovich C.P. (2016). Assessing Cancer Risk on Novel 29 MHz Micro-Ultrasound Images of the Prostate: Creation of the Micro-Ultrasound Protocol for Prostate Risk Identification. J. Urol..

[52] Rohrbach D., Wodlinger B., Wen J., Mamou J., Feleppa E. (2018). High-Frequency Quantitative Ultrasound for Imaging Prostate Cancer Using a Novel Micro-Ultrasound Scanner. Ultrasound Med. Biol..

[53] Tay K.J., Gupta R.T., Rastinehad A.R., Tsivian E., Freedland S.J., Moul J.W., Polascik T.J. (2016). Navigating MRI-TRUS Fusion Biopsy: Optimizing the Process and Avoiding Technical Pitfalls. Expert. Rev. Anticancer. Ther..

[54] Coker M.A., Glaser Z.A., Gordetsky J.B., Thomas J.V., Rais-Bahrami S. (2018). Targets Missed: Predictors of MRI-Targeted Biopsy Failing to Accurately Localize Prostate Cancer Found on Systematic Biopsy. Prostate Cancer Prostatic Dis..

[55] Klotz L., Andriole G., Cash H., Cooperberg M., Crawford E.D., Emberton M., Gomez-Sancha F., Klein E., Lughezzani G., Marks L. (2022). Optimization of Prostate Biopsy—Micro-Ultrasound versus MRI (OPTIMUM): A 3-Arm Randomized Controlled Trial Evaluating the Role of 29 MHz Micro-Ultrasound in Guiding Prostate Biopsy in Men with Clinical Suspicion of Prostate Cancer. Contemp. Clin. Trials.

[56] Org S., Cash H., Hofbauer S.L., Shore N., Pavlovich C.P., Bulang S., Schostak M., Planken E., Jaspars J.J., Luger F. (2022). Prostate Cancer Detection by Novice Micro-Ultrasound Users Enrolled in a Training Program. Soc. Int. Urol. J..

[57] Albers P., Bennett J., Evans M., Martin E.S., Wang B., Broomfield S., Martín A.M., Tu W., Fung C., Kinnaird A. (2024). Value of Incremental Biopsy Cores for Microultrasound Targeted Prostate Biopsies. Urology.

[58] Albers P., Wang B., Broomfield S., Medina Martín A., Fung C., Kinnaird A. (2022). Micro-Ultrasound Versus Magnetic Resonance Imaging in Prostate Cancer Active Surveillance. Eur. Urol. Open Sci..

[59] Kudlackova S., Kurfurstova D., Kral M., Hruska F., Vidlar A., Student V. (2021). Do Not Underestimate Anterior Prostate Cancer. Biomed. Pap. Med. Fac. Univ. Palacky Olomouc Czech Repub..

[60] Stamey T.A., Donaldson A.N., Yemoto C.E., Mcneal J.E., Sozen S., Gill H. (1998). Histological and Clinical Findings in 896 Consecutive Prostates Treated Only with Radical Retropubic Prostatectomy: Epidemiologic Significance of Annual Changes. J. Urol..

[61] McNeal J.E., Redwine E.A., Freiha F.S., Stamey T.A. (1988). Zonal Distribution of Prostatic Adenocarcinoma. Correlation with Histologic Pattern and Direction of Spread. Am. J. Surg. Pathol..

[62] McNeal J.E., Haillot O. (2001). Patterns of Spread of Adenocarcinoma in the Prostate as Related to Cancer Volume. Prostate.

[63] McNeal J.E. (1992). Cancer Volume and Site of Origin of Adenocarcinoma in the Prostate: Relationship to Local and Distant Spread. Hum. Pathol..

[64] Abouassaly R., Klein E.A., El-Shefai A., Stephenson A. (2020). Impact of Using 29 MHz High-Resolution Micro-Ultrasound in Real-Time Targeting of Transrectal Prostate Biopsies: Initial Experience. World J. Urol..

[65] Lughezzani G., Saita A., Lazzeri M., Paciotti M., Maffei D., Lista G., Hurle R., Buffi N.M., Guazzoni G., Casale P. (2019). Comparison of the Diagnostic Accuracy of Micro-Ultrasound and Magnetic Resonance Imaging/Ultrasound Fusion Targeted Biopsies for the Diagnosis of Clinically Significant Prostate Cancer. Eur. Urol. Oncol..

[66] Klotz L., Lughezzani G., Maffei D., Sánchez A., Pereira J.G., Staerman F., Cash H., Luger F., Lopez L., Shore N.D. (2020). Comparison of Micro-Ultrasound and Multiparametric Magnetic Resonance Imaging for Prostate Cancer: A Multicenter, Prospective Analysis. Can. Urol. Assoc. J..

[67] Lughezzani G., Maffei D., Saita A., Paciotti M., Diana P., Buffi N.M., Colombo P., Elefante G.M., Hurle R., Lazzeri M. (2021). Diagnostic Accuracy of Microultrasound in Patients with a Suspicion of Prostate Cancer at Magnetic Resonance Imaging: A Single-Institutional Prospective Study. Eur. Urol. Focus..

[68] Maffei D., Fasulo V., Avolio P.P., Saitta C., Paciotti M., De Carne F., Colombo P., Pasini L., De Zorzi S.Z., Saita A. (2023). Diagnostic Performance of MicroUltrasound at MRI-Guided Confirmatory Biopsy in Patients under Active Surveillance for Low-Risk Prostate Cancer. Prostate.

[69] Chessa F., Schiavina R., Amelio E., Gaudiano C., Giusti D., Bianchi L., Pultrone C., Marcelli E., Distefano C., Lodigiani L. (2021). Diagnostic Accuracy of the Novel 29 MHz Micro-Ultrasound “ExactVuTM” for the Detection of Clinically Significant Prostate Cancer: A Prospective Single Institutional Study. A Step Forward in the Diagnosis of Prostate Cancer. Arch. Ital. Urol. Androl..

[70] Cornud F., Lefevre A., Flam T., Dumonceau O., Galiano M., Soyer P., Camparo P., Barral M. (2020). MRI-Directed High-Frequency (29MhZ) TRUS-Guided Biopsies: Initial Results of a Single-Center Study. Eur. Radiol..

[71] Eure G., Fanney D., Lin J., Wodlinger B., Ghai S. (2019). Comparison of Conventional Transrectal Ultrasound, Magnetic Resonance Imaging, and Micro-Ultrasound for Visualizing Prostate Cancer in an Active Surveillance Population: A Feasibility Study. Can. Urol. Assoc. J..

[72] Staerman F. (2019). The Utility of 29 MHz Resolution Micro-Ultrasound and mpMRI in the Management of Gleason 6 Prostate Cancer with Active Surveillance. Eur. Urol. Suppl..

[73] García Garzón J.R., de Arcocha Torres M., Delgado-Bolton R., Ceci F., Alvarez Ruiz S., Orcajo Rincón J., Caresia Aróztegui A.P., García Velloso M.J., García Vicente A.M. (2018). 68Ga-PSMA PET/CT in Prostate Cancer. Rev. Esp. Med. Nucl. Imagen Mol..

[74] Farolfi A., Calderoni L., Mattana F., Mei R., Telo S., Fanti S., Castellucci P. (2021). Current and Emerging Clinical Applications of PSMA PET Diagnostic Imaging for Prostate Cancer. J. Nucl. Med..

[75] Malan N., Vangu M.D.T. (2022). Normal Variants, Pitfalls, and Artifacts in Ga-68 Prostate Specific Membrane Antigen (PSMA) PET/CT Imaging. Front. Nucl. Med..

[76] Piron S., Verhoeven J., Vanhove C., De Vos F. (2022). Recent Advancements in 18F-Labeled PSMA Targeting PET Radiopharmaceuticals. Nucl. Med. Biol..

[77] Veit-Haibach P., Ahlström H., Boellaard R., Delgado Bolton R.C., Hesse S., Hope T., Huellner M.W., Iagaru A., Johnson G.B., Kjaer A. (2023). International EANM-SNMMI-ISMRM Consensus Recommendation for PET/MRI in Oncology. Eur. J. Nucl. Med. Mol. Imaging.

[78] Giesel F.L., Hadaschik B., Cardinale J., Radtke J., Vinsensia M., Lehnert W., Kesch C., Tolstov Y., Singer S., Grabe N. (2017). F-18 Labelled PSMA-1007: Biodistribution, Radiation Dosimetry and Histopathological Validation of Tumor Lesions in Prostate Cancer Patients. Eur. J. Nucl. Med. Mol. Imaging.

[79] Dietlein M., Kobe C., Kuhnert G., Stockter S., Fischer T., Schomäcker K., Schmidt M., Dietlein F., Zlatopolskiy B.D., Krapf P. (2015). Comparison of [(18)F]DCFPyL and [ (68)Ga]Ga-PSMA-HBED-CC for PSMA-PET Imaging in Patients with Relapsed Prostate Cancer. Mol. Imaging Biol..

[80] Jetty S., Loftus J.R., Patel A., Gupta A., Puri S., Dogra V. (2023). Prostate Cancer—PET Imaging Update. Cancers.

[81] Alshamrani A.F.A. (2024). Diagnostic Accuracy of Molecular Imaging Techniques for Detecting Prostate Cancer: A Systematic Review. Diagnostics.

[82] Zhang H., Qi L., Cai Y., Gao X. (2024). Gastrin-Releasing Peptide Receptor (GRPR) as a Novel Biomarker and Therapeutic Target in Prostate Cancer. Ann. Med..

[83] Challapalli A., Barwick T.D., Dubash S.R., Inglese M., Grech-Sollars M., Kozlowski K., Tam H., Patel N.H., Winkler M., Flohr P. (2023). Bench to Bedside Development of [18F]Fluoromethyl-(1,2-2H4)Choline ([18F]D4-FCH). Molecules.

[84] Okudaira H., Shikano N., Nishii R., Miyagi T., Yoshimoto M., Kobayashi M., Ohe K., Nakanishi T., Tamai I., Namiki M. (2011). Putative Transport Mechanism and Intracellular Fate of Trans-1-Amino-3-18F-Fluorocyclobutanecarboxylic Acid in Human Prostate Cancer. J. Nucl. Med..

[85] Savir-Baruch B., Schuster D.M. (2022). Prostate Cancer Imaging with 18F-Fluciclovine. PET Clin..

[86] Robertson M.S., Sakellis C.G., Hyun H., Jacene H.A. (2020). Extraprostatic Uptake of 18F-Fluciclovine: Differentiation of Nonprostatic Neoplasms from Metastatic Prostate Cancer. Am. J. Roentgenol..

[87] Beer M., Montani M., Gerhardt J., Wild P.J., Hany T.F., Hermanns T., Müntener M., Kristiansen G. (2012). Profiling Gastrin-Releasing Peptide Receptor in Prostate Tissues: Clinical Implications and Molecular Correlates. Prostate.

[88] Gao X., Tang Y., Chen M., Li J., Yin H., Gan Y., Zu X., Cai Y., Hu S. (2023). A Prospective Comparative Study of [68Ga]Ga-RM26 and [68Ga]Ga-PSMA-617 PET/CT Imaging in Suspicious Prostate Cancer. Eur. J. Nucl. Med. Mol. Imaging.

[89] Schollhammer R., De Clermont Gallerande H., Yacoub M., Quintyn Ranty M.L., Barthe N., Vimont D., Hindié E., Fernandez P., Morgat C. (2019). Comparison of the Radiolabeled PSMA-Inhibitor 111In-PSMA-617 and the Radiolabeled GRP-R Antagonist 111In-RM2 in Primary Prostate Cancer Samples. EJNMMI Res..

[90] Rowe S.P., Pienta K.J., Pomper M.G., Gorin M.A. (2018). PSMA-RADS Version 1.0: A Step Towards Standardizing the Interpretation and Reporting of PSMA–Targeted PET Imaging Studies. Eur. Urol..

[91] Emmett L., Buteau J., Papa N., Moon D., Thompson J., Roberts M.J., Rasiah K., Pattison D.A., Yaxley J., Thomas P. (2021). The Additive Diagnostic Value of Prostate-Specific Membrane Antigen Positron Emission Tomography Computed Tomography to Multiparametric Magnetic Resonance Imaging Triage in the Diagnosis of Prostate Cancer (PRIMARY): A Prospective Multicentre Study. Eur. Urol..

[92] Uprimny C., Kroiss A.S., Decristoforo C., Fritz J., von Guggenberg E., Kendler D., Scarpa L., di Santo G., Roig L.G., Maffey-Steffan J. (2017). 68Ga-PSMA-11 PET/CT in Primary Staging of Prostate Cancer: PSA and Gleason Score Predict the Intensity of Tracer Accumulation in the Primary Tumour. Eur. J. Nucl. Med. Mol. Imaging.

[93] Demirci E., Kabasakal L., Şahin O.E., Akgün E., Gültekin M.H., Doǧanca T., Tuna M.B., Öbek C., Kiliç M., Esen T. (2019). Can SUVmax Values of Ga-68-PSMA PET/CT Scan Predict the Clinically Significant Prostate Cancer?. Nucl. Med. Commun..

[94] Seifert R., Emmett L., Rowe S.P., Herrmann K., Hadaschik B., Calais J., Giesel F.L., Reiter R., Maurer T., Heck M. (2023). Second Version of the Prostate Cancer Molecular Imaging Standardized Evaluation Framework Including Response Evaluation for Clinical Trials (PROMISE V2). Eur. Urol..

[95] Yechiel Y., Orr Y., Gurevich K., Gill R., Keidar Z. (2023). Advanced PSMA-PET/CT Imaging Parameters in Newly Diagnosed Prostate Cancer Patients for Predicting Metastatic Disease. Cancers.

[96] Kase A.M., Tan W., Copland J.A., Cai H., Parent E.E., Madan R.A. (2022). The Continuum of Metastatic Prostate Cancer: Interpreting PSMA PET Findings in Recurrent Prostate Cancer. Cancers.

[97] Mütevelizade G., Sezgin C., Gümüşer G., Sayit E. (2022). Unexpected Metastatic Localizations of Prostate Cancer Determined by 68Ga PSMA PET/CT: Series of Four Cases. Mol. Imaging Radionucl. Ther..

[98] Afshar-Oromieh A., Holland-Letz T., Giesel F.L., Kratochwil C., Mier W., Haufe S., Debus N., Eder M., Eisenhut M., Schäfer M. (2017). Diagnostic Performance of 68Ga-PSMA-11 (HBED-CC) PET/CT in Patients with Recurrent Prostate Cancer: Evaluation in 1007 Patients. Eur. J. Nucl. Med. Mol. Imaging.

[99] Liu F.Y., Sheng T.W., Tseng J.R., Yu K.J., Tsui K.H., Pang S.T., Wang L.J., Lin G. (2022). Prostate-Specific Membrane Antigen (PSMA) Fusion Imaging in Prostate Cancer: PET-CT vs PET-MRI. Br. J. Radiol..

[100] Chow K.M., So W.Z., Lee H.J., Lee A., Yap D.W.T., Takwoingi Y., Tay K.J., Tuan J., Thang S.P., Lam W. (2023). Head-to-Head Comparison of the Diagnostic Accuracy of Prostate-Specific Membrane Antigen Positron Emission Tomography and Conventional Imaging Modalities for Initial Staging of Intermediate- to High-Risk Prostate Cancer: A Systematic Review and Meta-Analysis. Eur. Urol..

[101] Huo H., Shen S., He D., Liu B., Yang F. (2022). Head-to-Head Comparison of 68Ga-PSMA-11 PET/CT and 68Ga-PSMA-11 PET/MRI in the Detection of Biochemical Recurrence of Prostate Cancer: Summary of Head-to-Head Comparison Studies. Prostate Cancer Prostatic Dis..

[102] Santiago Almeida L., Etchebehere E.C.S.d.C., García Megías I., Calapaquí Terán A.K., Hadaschik B., Colletti P.M., Herrmann K., Giammarile F., Delgado Bolton R.C. (2024). PSMA Radioligand Therapy in Prostate Cancer Where Are We and Where Are We Heading?. Clin. Nucl. Med..

[103] Sartor O., de Bono J., Chi K.N., Fizazi K., Herrmann K., Rahbar K., Tagawa S.T., Nordquist L.T., Vaishampayan N., El-Haddad G. (2021). Lutetium-177–PSMA-617 for Metastatic Castration-Resistant Prostate Cancer. N. Engl. J. Med..

[104] Morris M.J., Castellano D., Herrmann K., de Bono J.S., Shore N.D., Chi K.N., Crosby M., Piulats J.M., Fléchon A., Wei X.X. (2024). 177Lu-PSMA-617 versus a Change of Androgen Receptor Pathway Inhibitor Therapy for Taxane-Naive Patients with Progressive Metastatic Castration-Resistant Prostate Cancer (PSMAfore): A Phase 3, Randomised, Controlled Trial. Lancet.

[105] Pepe P., Pepe L., Tamburo M., Marletta G., Pennisi M., Fraggetta F. (2022). Targeted Prostate Biopsy: 68Ga-PSMA PET/CT vs. MpMRI in the Diagnosis of Prostate Cancer. Arch. Ital. Urol. Androl..

[106] Lopci E., Saita A., Lazzeri M., Lughezzani G., Colombo P., Buffi N.M., Hurle R., Marzo K., Peschechera R., Benetti A. (2018). 68Ga-PSMA Positron Emission Tomography/Computerized Tomography for Primary Diagnosis of Prostate Cancer in Men with Contraindications to or Negative Multiparametric Magnetic Resonance Imaging: A Prospective Observational Study. J. Urol..

[107] Pepe P., Pepe L., Tamburo M., Marletta G., Savoca F., Pennisi M., Fraggetta F. (2023). 68Ga-PSMA PET/CT Evaluation in Men Enrolled in Prostate Cancer Active Surveillance. Arch. Ital. Urol. Androl..

[108] Krausewitz P., Bundschuh R.A., Gaertner F.C., Essler M., Attenberger U., Luetkens J., Kristiansen G., Muders M., Ohlmann C.H., Hauser S. (2023). DEPROMP Trial: The Additive Value of PSMA-PET/CT-Guided Biopsy for Prostate Cancer Management in Biopsy Naïve Men-Study Protocol for a Randomized Trial. Trials.

[109] Lopci E., Lazzeri M., Colombo P., Casale P., Buffi N.M., Saita A., Peschechera R., Hurle R., Marzo K., Leonardi L. (2023). Diagnostic Performance and Clinical Impact of PSMA PET/CT versus MpMRI in Patients with a High Suspicion of Prostate Cancer and Previously Negative Biopsy: A Prospective Trial (PROSPET-BX). Urol. Int..

[110] Gondoputro W., Doan P., Katelaris A., Scheltema M.J., Geboers B., Agrawal S., Liu Z., Yaxley J., Savdie R., Rasiah K. (2023). 68Ga-PSMA-PET/CT in Addition to MpMRI in Men Undergoing Biopsy during Active Surveillance for Low- to Intermediate-Risk Prostate Cancer: Study Protocol for a Prospective Cross-Sectional Study. Transl. Androl. Urol..

[111] Krausewitz P., Gaertner F.C., Essler M., Attenberger U., Luetkens J., Kristiansen G., Ohlmann C.H., Hauser S., Ellinger J., Ritter M. (2024). DEPROMP-Study: PSMA-PET/CT Prior to Prostate Biopsy: Enhancing Prostate Cancer Detection and Personalized Management. Eur. Urol..

[112] Mookerji N., Pfanner T., Hui A., Huang G., Albers P., Mittal R., Broomfield S., Dean L., St Martin B., Jacobsen N.-E. (2024). Fluorine-18 Prostate-Specific Membrane Antigen–1007 PET/CT vs Multiparametric MRI for Locoregional Staging of Prostate Cancer. JAMA Oncol..

[113] Liu C., Liu T., Zhang Z., Zhang N., Du P., Yang Y., Liu Y., Yu W., Li N., Gorin M.A. (2020). 68Ga-PSMA PET/CT Combined with PET/Ultrasound-Guided Prostate Biopsy Can Diagnose Clinically Significant Prostate Cancer in Men with Previous Negative Biopsy Results. J. Nucl. Med..

[114] Lopci E., Lazzeri M., Disconzi L., Colombo P., Saita A., Peschechera R., Fasulo V., Maffei D., Zanca R., Casale P. (2024). Interim Analyses from the PROSPET-BX Trial: [G8Ga]PSMA PET/CT vs. MpMRI in Patients with Suspicion of Prostate Cancer and Previous Negative Biopsy. J. Clin. Oncol..

[115] Jain A., Nassour A.J., Dean T., Patterson I., Tarlinton L., Kim L., Woo H. (2023). Expanding the Role of PSMA PET in Active Surveillance. BMC Urol..

